# The matrix metalloproteinase 7 (MMP7) links Hsp90 chaperone with acquired drug resistance and tumor metastasis

**DOI:** 10.1002/cnr2.1261

**Published:** 2020-08-06

**Authors:** Pankaj Kumar, Satish Siripini, Amere Subbarao Sreedhar

**Affiliations:** ^1^ CSIR‐Centre for Cellular and Molecular Biology Hyderabad India; ^2^ Presently at IMT College of Pharmacy Puri India

**Keywords:** Cancer, drug resistance, Hsp90, metastasis, MMP7

## Abstract

**Background:**

Cancer emergence is associated with a series of cellular transformations that include acquired drug resistance followed by tumor metastasis. Matrix metalloproteinases (MMPs) and Hsp90 chaperone are implicated in tumor progression, however, they are not studied in the context of drug resistance.

**Aims:**

In the present study, we aimed at understanding the cross‐talk between acquired drug resistance and tumor progression, linking MMP7 and Hsp90.

**Methods and results:**

We have developed an *in vitro* model system for acquired drug resistance and studied the correlation between MMP7 and Hsp90. We demonstrate that enhanced drug efflux activity correlates with the induced expression and activity of MMP7, and enhanced metastatic potential of cells, however, in Hsp90‐dependent manner. The MMP7 overexpression alone could enhance the drug efflux activity marginally, and metastasis significantly. However, challenging these cells with 17AAG has significantly increased the drug efflux activity and, in contrast, decreased the metastatic potential. Evaluating our *in vitro* findings in mice xenografts revealed that MMP7 overexpression facilitates altered homing properties. However, these cells, in response to 17AAG treatment, exhibited increased localized tumor growth but decreased tumor metastasis.

**Conclusion:**

We demonstrated a cross‐talk between Hsp90 and MMP7 in regulating the acquired drug resistance and tumor progression. Our findings provide novel insights on targeting drug resistant‐tumors.

## Graphical abstract

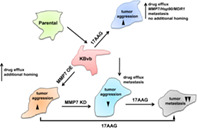

## 1 INTRODUCTION

The cancer emergence is a polygenic phenomenon and involves continuous remodeling and adaptation of cells to its microenvironment. The matrix metalloproteinases (MMPs) are the endopeptidases from the metzincin family involved in the degradation of extracellular matrix (ECM) proteins during organogenesis, growth, and normal tissue turnover.^1^ Due to their broad substrate specificity, MMPs are implicated in several biological processes that include angiogenesis, immunity and wound healing.^2^, ^3^ While tissue remodeling has considered to be a developmentally regulated phenomenon, cancer cells make use of it for their survival, propagation, and disease progression.^4^ There are 25 MMPs reported in humans with diverse substrate specificities and structural differences. Despite having broad substrate specificity, MMPs are implicated in cancer progression.^5^

The multidrug resistance facilitates the efflux of drugs and toxic compounds by the cells.^6^ The acquired drug resistance allows tumor cells to overcome therapeutic insults, thus promotes tumor aggression. The drug resistance majorly mediated by P‐glycoprotein (P‐gp) and a subset of multidrug resistance proteins (MRPs) both belong to the ATP‐binding cassette (ABC) family of transporters.^7^, ^8^ Linking tumor evolution with drug resistance and metastasis has been a long‐standing debate. However, it is agreed that both the cellular processes coordinately function to promote tumor survival, spread, and progression. Till date, no direct correlation is being demonstrated between drug resistance with tumor metastasis by any of these studies.^9^, ^10^, ^11^

The cancer chaperone, Hsp90 has been implicated in tumor progression, especially in promoting the proliferative potential of cells through stabilizing the functions of mutated kinases.^12^ Hsp90 also implicated in tumor cell migration, invasion, and angiogenesis.^13^, ^14^, ^15^ While the small molecular weight heat shock proteins (Hsps) such as Hsp27 and Hsp70 are implicated in drug resistance, their role in tumor metastasis is less understood.^16^, ^17^ However, Hsp90 is extensively studied for its role in tumor metastasis, but not in drug resistance.^18^, ^19^ Hsp90 contributes to tumor metastasis through stabilizing the functions of MMPs such as MMP2, and MMP9.^20^, ^21^ Both MMP2 and MMP9 are extensively studied for cancer metastasis in a large variety of experimental as well as clinical cancer models, therefore, inhibiting, MMPs is suggested to combat cancer.^22^

Earlier, we demonstrated that Hsp90 plays a role in the acquired multidrug resistance of cancer cells.^23^ By extrapolating these studies, we asked whether there is any cross‐talk between Hsp90 and MMP7 in regulating the drug resistance and tumor metastasis. The MMP7 can act upstream of MMP2 and MMP9, and similar to Hsp90 also overexpressed in highly metastatic cells.^24^ While studying the effects of Hsp90 inhibition against the drug‐resistant tumor cells, we have come across MMP7 interference with the therapeutic response mediated by Hsp90 inhibitor, 17AAG. Therefore, we have examined the functional consequence of MMP7 expression in regulating the drug resistance and tumor metastasis. Our findings provide novel insights linking Hsp90 with MMP7 in regulating these two mechanisms, thus may have clinical implications.

## 2 MATERIALS AND METHODS

### 2.1 Cell culture maintenance and drug treatments

The KB cells (ATCC‐CCL‐17), HeLa derivative, were procured from American Type Culture Collection (ATCC), authenticated using short tandem repeat (STR) DNA profiling at CSIR‐CCMB and tested for contamination from mycoplasma (DAPI staining), bacteria (medium turbidity), and yeast (appearance of bead‐like outgrowth in the culture plate correlating with turbidity) before using. Cells maintained in DMEM (GIBCO, #12100‐061) containing 10% FBS (GIBCO, #16000‐044) in the presence of penicillin, streptomycin, and kanamycin at 37°C in a humidified incubator with 5% CO_2_ supply. The 2D culture cells were grown on a plastic surface (NUNC), and for 3D cultures, the plastic surface coated with matrigel (BD Biosciences, #354234) before plating the cells. The cells (0.2 × 10^6^) grown in a 6‐well culture dishes (Thermo Scientific, #140675) and a final concentration of 2.0 μM 17AAG (#ant‐agl‐25, Invivogen) was used for treatments based on our earlier studies.^25^ Quercetin at a final concentration of 10 μM (#Q4951‐10G, Sigma‐Aldrich) was used to inhibit the stress response.^26^ The drug‐resistant cells were prepared by treating KB Parental cells with lower to higher concentrations of vinblastine (0.5–2.0 μM, Sigma‐Aldrich, V1377‐5MG), and its combination with cisplatin (0.5–2.0 μM, Sigma‐Aldrich, C2210000) for 60 passages and named KBvb and KBvbcp (drug‐resistant), respectively.^23^

### 2.2 Doubling time calculation

The doubling time is the time required for cells to double in number. The doubling time was calculated using the formula: DT = *T* ln2/ln(Xe/Xb), where “*T*” is incubation time, “Xb” is initial cell number, “Xe” is the cell number at the end of the incubation time. (https://www.atcc.org/~/media/PDFs/Culture%20Guides/AnimCellCulture_Guide.ashx).

### 2.3 RNA isolation, reverse transcriptase polymerase chain reaction (RT‐PCR)

The total RNA isolated from the cells using Trizol (Invitrogen, #15596018). First strand cDNA was synthesized from 1 μg of total RNA using the Primescript cDNA synthesis kit (Takara Clontech, #6110A). The various gene expression levels were examined by RT‐PCR using gene specific primers and using 10 ng of cDNA. The primers used are, *MMP7* (NM_002423.3), forward primer—5′‐GGAACAGGCTCAGGACTATCTC‐3′ and reverse primer—5′‐GTCAGCAGTTCCCCATACAA‐3′, *CCND1* (NM_053056.2), forward primer—5′‐AACTACCTGGACCGCTTCCT‐3′ and forward primer—5′‐TGAGGCGGTAGTAGGACAGG‐3′, *HSP90α* (NM_001017963.2), forward primer—5′‐ACCAAAGAAGGCCTGGAACT‐3′ and reverse primer—5′‐TTCTTCCATGCGTGATGTGT‐3′, *HSP90β* (NM_001271969.1), forward primer—5′‐ATGCTCCAGCAGAGCAAAAT‐3′ and reverse primer—5′‐GCAGCAAGGTGAAGACACAA‐3′, *Hsp70* (NM_005346.6), forward primer—5′‐GGATGCGGCCAAGAACCAGGTG‐3′ and reverse primer—5′‐CCCGTTCTGTCCAGGCCGTAGG‐3′, *GAPDH* (NM_002046.5), forward primer—5′‐CCTCCCGCTTCGCTCTCTGCT‐3′ and reverse primer—5′‐TGCAAATGAGCCCCAGCCTCC‐3′, *SNAIL* (NM_005985.3), forward primer—5′‐GCCTCGCTGCCAATGCT‐3′ and reverse primer—5′‐TGTTTGGGTCGGTCTGGATG‐3′, *N‐Cadherin* (NM_001308176.1), forward primer—5′‐CAGGTTTGGAATGGGACAGTT‐3′ and reverse primer—5′‐TGTTTGGGTCGGTCTGGATG‐3′, *HIF1α* (NM_001530.3), forward primer—5′‐CGTCGCTTCGGCCAGTGTGT‐3′, reverse primer—5′‐TGGTGAATCGGTCCCCGCGA‐3′, *VEGF‐A* (NM_001287044.1), forward primer—5′‐CGAAGTGGTGAAGTTCATGGATG‐5′ and reverse primer—5′‐TTCTGTATCAGTCTTTCCTGGTGAG‐3′, *MUC1* (NM_002456.5), forward primer—5′‐TGGCTGTCTGTCAGTGC‐3′ and reverse primer—5′‐CTACAAGTTGGCAGAAGTGG‐3′, *VIMENTIN* (NM_003380.3), forward primer—5′‐GGACCAGCTAACCAACGACA‐3′ and reverse primer—5′‐ACCATTCTTCTGCCTCCTGC‐3′, *TWIST* (NM_000474.3), forward primer—5′‐TTCTCGGTCTGGAGGATGGA‐3′ and reverse primer—5′‐CAGAGGTGTGAGGATGGTGC‐3′, *TJP1* (NM_003257.4), forward primer—5′‐TACAACTGGGGGAGGGTGAA‐3′ and reverse primer—5′‐CAAACAGACCAAGCCAGCAC‐3′, *CYTOKERATIN 18* (NM_000224.2), forward primer—5′‐CAAAGCCTGAGTCCTGTCCT‐3′ and reverse primer—5′‐TGGCAATCTGGGCTTGTAGG‐3′, *HES‐1* (NM_005524.3), forward primer—5′‐CCTCGTCCCCGGTGGCTGCT‐3′ and reverse primer—5′‐GCCGTCATCTGCGCCCGCTG‐3′, *HEY‐1* (NM_012258.3), forward primer—5′‐TTGAGAAGCGCCGACGAGAC‐3′ and reverse primer—5′‐CGAACTCGAAGCGGGTCAGA‐3′, *CD44* (NM_000610.3), forward primer—5′‐ACAATGGCCCAGATGGAGAA‐3′ and reverse primer—5′‐CCGTGGTGTGGTTGAAATGG‐3′, *CD24* (NM_013230.3), forward primer—5′‐CTGCTGGCACTGCTCCTA‐3′ and reverse primer—5′‐CCTTGGTGGTGGCATTAGTT‐3′.

### 2.4 Quantitative PCR analysis

The quantitative PCR was performed from the cDNA obtained in Section 2.3. The primers used for the quantitative PCR analysis (ABI Biosystems) are, *GAPDH* (NM_002046.5), forward primer—5′‐TTGCCATCAATGACCCCTTCA‐3′ and reverse primer—5′‐CGCCCCACTTGATTTTGGA‐3′, *MDR1* (NM_001348945.1), forward primer—5′‐GCTGGTTGCTGCTTACATTCA‐3′ and reverse primer—5′‐CCAACATCGTGCACATCAAA‐3′, *Hsp90α* (NM_005348.4), forward primer—5′‐CTGCACCAGAATGAAGGAGA‐3′ and reverse primer—5′‐GAAGACGTTCCACAAAGGCT‐3′, *Hsp90β* (NM_001371238.1), forward primer—5′‐GCAGACATCTCCATGATTGG‐3′ and reverse primer—5′‐AAGGAACCTCCAGCAGAAGA‐3′, *MMP7* (NM_002423.4), forward primer—5′‐TACAGTGGGAACAGGCTCA‐3′ and reverse primer—5′‐GCATCTCCTTGAGTTTGGCT‐3′.

### 2.5 Fluorescence‐activated cell sorting (FACS) analysis

To obtain the survival fitness as well as to measure the DNA content of cells, after respective treatments, cells were treated with 250 nM Calcein (Invitrogen, #V13180) and Propidium Iodide (PI, 10 μg/mL, Calbiochem, #537059‐250MG) for live *vs* dead cell scoring and analyzed in a flow cytometer (BD FacsCalibur, CA). For DNA content analysis, cells after respective treatments were harvested in PBS by gentle scraping and fixed in ice‐cold ethanol (70%) for 15 minutes. Cells stained with PI containing RNase A (10 μg/mL, Thermo Scientific, # ENO531) were analyzed by FACS.

### 2.6 Immunoblot analysis

Cells were lysed into RIPA buffer (Radioimmunoprecipitation assay buffer, pH 8.0) and incubated at 4°C for 1 hour with gentle agitation. The lysates were clarified, and protein concentrations were estimated by the Bradford method. The lysates (30 μg) were resolved on SDS‐PAGE (10%) and transferred onto the nitrocellulose membrane. The membrane was blocked with 5% BSA in TBST and incubated with primary antibodies to MMP7 (1:2000; ab176325, Abcam), Hsp90α (1:1000; GTX60587; Genetex) and GAPDH (1:1000; SC‐32233, Santacruz Biotechnologies) and HRP‐conjugated secondary antibodies (1:10 000; 11500694001, Sigma‐Aldrich), each for 1 hour at room temperature. The luminescence was detected by the BM chemiluminescence detection kit (Roche, #11520709001).

### 2.7 The in‐gel activity assays

#### 2.7.1 Casein zymography

Cells (1 × 10^5^) were plated in 35 mm NUNC plate, and after 6 hours, the complete medium was replaced with serum‐free medium and incubated further for 24 hours. An in‐house procedure used to remove albumin from the medium, and the conditioned medium was subjected to freeze dry. For the in‐gel assay, 12% SDS‐polyacrylamide gel (SDS‐PAGE) was prepared with Bovine β‐casein (0.5 mg/mL; C6905‐1G, Sigma‐Aldrich). The pre‐run was done at room temperature at a constant current (40 mA) for 30 minutes. The Laemilli buffer (1×) reducing agents was added to the sample (30 μL), boiled, and loaded on to the gel. The electrophoresis was performed at constant current (20 mA at 4°C). The gel was washed twice with 2.5% Triton X‐100 and 50 mM Tris‐Cl (pH 7.5). Then the gel was washed twice with 50 mM Tris‐Cl buffer, for 10 minutes and incubated at 37°C overnight in developing buffer (0.15 M NaCl, 10 mM CaCl_2_, 0.1% Triton X‐100, 0.02% NaN_3_, 50 mM Tris‐Cl, pH 7.5). Subsequently, the gel was stained (with 0.5% Coomassie Brilliant Blue R 250 in 10% acetic acid), destained with 10% acetic acid until the clean bands were visualized.

#### 2.7.2 Gelatin zymography

To the 30 μL sample prepared from the conditioned medium, Laemilli buffer (1×) reducing agents was added the sample (30 μL), boiled, and loaded on to the gel. SDS‐polyacrylamide gel (10%) was polymerized with 0.1% gelatin (G9136‐10MG, Sigma‐Aldrich). The gel run was performed at a constant voltage (100 V) for one and a half hours. Then the gel was washed with (2.5%, ×2) Triton X‐100 for 30 minutes to remove SDS. Further, the gel was incubated at 37°C for overnight in the developing buffer (0.15 M NaCl, 10 mM CaCl_2_, 0.5 mM ZnCl_2_, 50 mM Tris‐Cl, pH 7.5). The gel was stained (with 0.5% Coomassie Brilliant Blue R 250 in 10% acetic acid), destained with 10% acetic acid until the clean bands were visualized.

### 2.8 Rhodamine 123 efflux assay

Cells (4 × 10^5^) grown in a six‐well culture dish for 6 hours before respective treatments (like‐17AAG and quercetin for 24 hours) and were washed with PBS, incubated with 1 μM concentration of Rhodamine 123 (Rh123, #R302, Thermo Fisher Scientifics) in the incomplete medium at 37°C for 30 minutes. After the incubation, cells were washed with PBS, and the incomplete medium was added, another set was incubated with 5 μM of cyclosporine A (#30024‐25MG, Sigma‐Aldrich) for 2 hours to examine the maximum drug accumulation. Cells were washed with 1× PBS and scraped into 1 mL of PBS on ice and analyzed using a flow cytometer (Beckman Coulter, Gallios). The efflux ratio was calculated by the mean channel value of Cyclosporin A/mean channel value of treatments as per our previous publication.^23^

### 2.9 Construction of MMP7 overexpression (MMP7 OE) system

Full‐length human *MMP7* cDNA (NM_002423.5) was amplified using gene‐specific primers from the cDNA library prepared from the Parental KB cells using forward primer—5′‐CGGAATTCCGATGCGACTCACCGTGCTG‐3′ and reverse primer—5′‐CGGGATCCCGCTATTTCTTTCTTGAATTACTTCTCTTT‐3′, where the primers are containing EcoRI and BamH1 restriction sites respectively. The PCR product was digested with restriction enzymes and gel purified. Similarly, the pEGFP‐C1‐FLAG was received from Dr Veena K. Parnaik at CCMB (Addgene plasmid #46956; http://n2t.net/addgene:46956; RRID:Addgene_46 956)^27^ was digested and gel purified. The vector and insert were used in a 1:3 M ratio for ligation. After the screening by restriction digestion as well as by automated Sanger's dideoxy DNA sequencing (model: ABI 3730) the recombinant plasmid was used for transfections (Figure S1).

### 2.10 Construction of MMP7 knockdown (MMP7 KD) system

The MMP7 knockdown (NM_002423.5) was achieved by the shRNA approach, where the shRNA was designed with the help of InvivoGen siRNA wizard software. To knockdown MMP7, we custom synthesized the forward—5′‐CCGGGGACTATCTCAAGAGTTTCTCGAGAAATCTCTTGAGATAGTCCTTTTTG‐3′ and reverse—5′‐AATTCAAAAAGGACTATCTCAAGAGATTTCTCGAGAAATCTCTTGAGATAGTCC‐3′ oligos and cloned into PLKO.1 TRC cloning system (pLKO.1‐TRC cloning vector (Addgene plasmid #10878; http://n2t/addgene:10878; RRID:Addgene_10878).^28^ After obtaining the recombinant plasmid, shRNA cloning was confirmed by automated Sanger's dideoxy DNA sequencing (model: ABI3730), and cells were transfected with shRNA plasmid (Figure S2), selected with antibiotics and used.

### 2.11 Transfection by Lipofectamine 3000

Transfection of MMP7 OE and MMP7 KD plasmids were performed as per the manufacturers' instruction. In brief, the 4 × 10^5^ cells per well were plated into six‐well culture plate, after 5 hours the Lipofectamine and 2 μg plasmid DNA were mixed in serum‐free medium and incubated at room temperature for 10 minutes. The media was removed from the cells and 600 μL fresh complete medium was added. The lipid‐DNA complex was added drop by drop and mixed thoroughly by shaking the plates. Cells were incubated at standard culture conditions for 5 hours, and the media was replaced with complete medium. After 24 hours, particular antibiotics were added for the stable selection and continued for 21 days.

### 2.12 Angiogenesis assay

The angiogenesis assay or the tube formation ability of cells is determined by the formation of branching of cells on 3D cultures. Before plating the cells, the six‐well NUNC plates were coated with 100 μL matrigel (BD Biosciences, #354234). The plates were then kept in an incubator at 37°C for 30 minutes. After incubation, cells (0.4 × 10^6^/well) were placed on matrigel coated surface, and the ability of cells to form tube‐like structures was examined after 24 hours under the live cell imaging microscope (Axiovert 200M). The number of branch points indicates high angiogenic potential, hence the number of branch points was calculated manually from five different replicates and represented.

### 2.13 Wound healing assay

Cells were grown as a monolayer (90% confluence) in a 35 mm dish (NUNC, #153066). With the help of a sterile yellow tip, a scratch (wound) was made on the monolayer of cells, the wounded area was cleared, and the area was measured before and after 48 hours of 17AAG treatment (Axiovert 200M, Zeiss, Oberkochen, Germany).

### 2.14 Tumor invasion assay

The invasive potential of cells was examined by transwell (Corning, #CLS3464‐12EA) migration assay. The lower chamber was filled with the complete medium while the cells are placed in the upper chamber coated with 50 μL of matrigel in serum‐free media. Cells invaded to the lower chamber by 24 hours incubation at standard culture conditions were stained with crystal violet (0.1% in sterile double distilled water) and observed under the microscope (Axiovert 200 M). The number of cells in the lower compartment at a defined time interval was counted and converted into % cell invasion and represented.

### 2.15 Animal ethics statement and mice xenograft models

This study was carried out as per the guidelines and recommendations of the National Institute of Health (NIH). The animal handling procedures were approved by the Institutional Animal Ethics Committee (Project No. IAEC 01/2017). Each experiment was done a minimum three times, having six animals in each group. Male nude mice were maintained with standard diet and water in ventilated cages under controlled conditions of temperature and humidity. Six‐week‐old male nude mice were injected with Parental and KBvb OE cells (5 × 10^6^) in 50 μL PBS with 50 μL matrigel either alone or after 17AAG treatments intramuscularly in plank region. Mice were monitored for s.c. tumor growth over a period (5 weeks), and care was taken in such a way that the tumor volume is always maintained within the accepted range of 2‐5 cm. Subsequently, animals after 5 weeks were euthanized, and different tissues were dissected and embedded in paraffin using standard approved institutional procedures. The paraffin‐embedded tissue sections were stained with Hematoxylin (Sigma‐Aldrich, #H9627) and Eosin (Sigma‐Aldrich, #230251) and observed under the microscope (Axioimager.Z2, Zeiss).

### 2.16 Statistical analysis

The data represented are from three independent experiments mean ± SD. The statistical significance is calculated from Student's *t*‐test for Figure 3 and one‐way ANOVA and Tukey test for other Figures. The choice of statistics was based on data representation, where ever two groups were used, we chose Student *t* test, and where ever more than two groups were used, we chose one‐way ANOVA.

**FIGURE 1:**
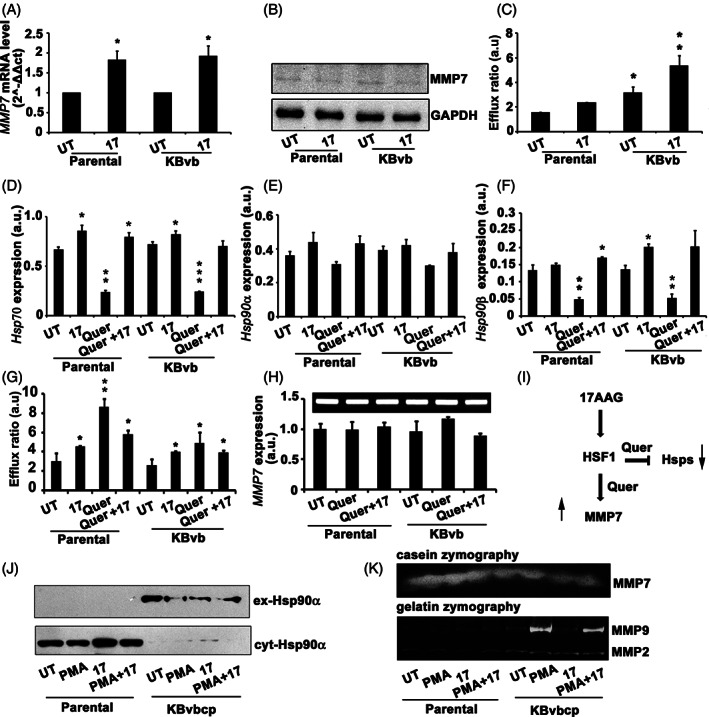
17AAG treatment induces MMP7 expression, drug efflux activity and, heat shock response. A, Quantitative PCR analysis of *MMP7* expression in untreated and 17AAG treated Parental and KBvb cells. B, Immunoblot analysis of MMP7 levels from the total cell lysates. GAPDH was used as a loading control. C, Rh123 efflux activity assay of Parental and KBvb cells in response to 17AAG treatment. Note 17AAG treatment inducing the drug efflux activity in the sensitive and drug‐resistant cells. D‐F, Effect of quercetin on inhibiting the stress response induced by 17AAG was examined by the transcript levels of *Hsp70*, *Hsp90α*, and *Hsp90β*. Note quercetin treatment decreasing 17AAG induced stress response. G, Rh123 efflux activity assay showing the effect of quercetin on drug efflux activity. H, RT‐PCR analysis of *MMP7* expression in response to quercetin treatment. Note quercetin not having an influence on *MMP7* transcription. I, Schematic representation showing quercetin influence only on *Hsp* expression, but not on *MMP7* expression. J, Immunoblot analysis of Hsp90α in total cell lysates (cyt‐Hsp90α) as well as from the conditioned medium (ex‐Hsp90α). K, In‐gel activity assay for MMP7 (casein zymography), MMP2, and MMP9 (gelatin zymography). Note induced activity of MMP7 correlating with ex‐Hsp90α. Parental: KB cells; KBvb: KB vinblastine resistant; KBvbcp: KB cells resistant to vinblastine and cisplatin; UT: untreated; 17: 17AAG: Quer: quercetin; cyt: cytoplasm; ex: extracellular. The values (mean ± SD) represented in figures, A, C and D‐H are average values obtained from three independent experiments. The significance values calculated from one‐way ANOVA and represented as, *P < 0.05, **P < 0.01, ***P < 0.001

**FIGURE 2:**
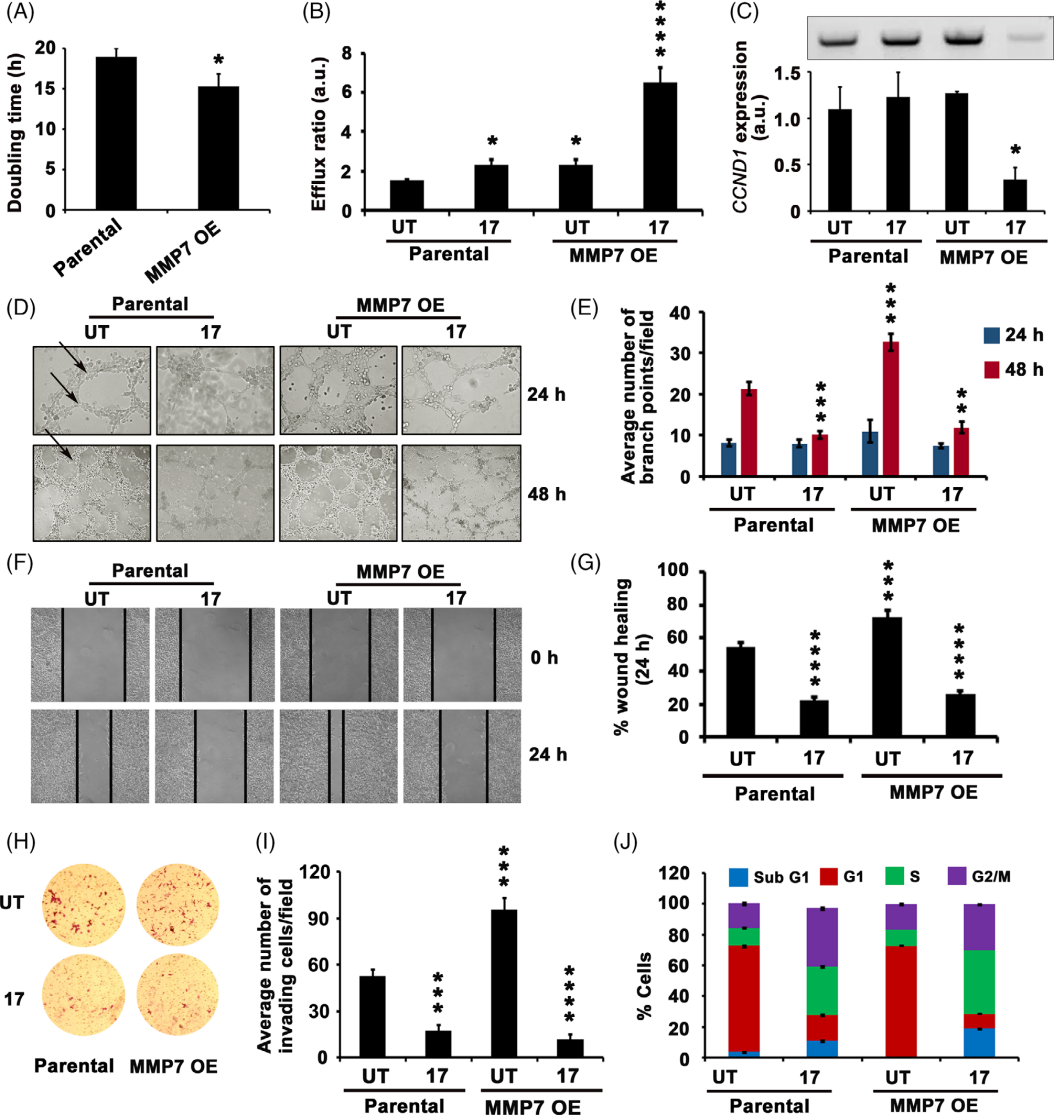
The MMP7 overexpression (OE) potentiating the cell proliferation, drug efflux activity and metastatic potential. A, Calculation of doubling time between Kbvb and Kbvb‐MMP7 OE cells. Note MMP7 OE inducing cell proliferation. B, Rh123 efflux activity assay of Kbvb and KBvb‐MMP OE cells. Note MMP7 OE itself inducing the efflux activity of cells, which is furthered with 17AAG treatment. C, RT‐PCR analysis of *CCND1* expression, a proliferation marker. Note that MMP7 OE cells are sensitive to 17AAG induced proliferation block despite showing enhanced efflux activity. D, Analysis of angiogenic potential (tube forming ability) of cells on 3D matrix. Note MMP7 OE increasing the branch points. E, Statistical representation of D. F, The in vitro scratch would heal assay for Parental and MMP7 OE cells. G, We have compared wound healing after 24 hours with 0 hour and calculated percent wound healing. F and H, The trans‐well migration assay to understand the invasive potential of MMP7 OE on cells in comparison with Parental cells. Note that MMP7 OE cells showing enhanced invasion. I, A statistical representation of H. J, DNA content analysis of Parental and MMP7 OE cells. Cells with 2n DNA content are G1‐phase cells, cells with 4n DNA content are G2/M phase cells, the cells in between 2n and 4n DNA content are S‐phase cells. Cells not in any cycling phase are subG1 cells. Parental: KB cells; KBvb: KB vinblastine resistant; UT: untreated; 17: 17AAG. The values (mean ± SD) represented in figures, A, E, G, I, and J are average values obtained from three independent experiments. The significance values calculated from one‐way ANOVA and represented as, *P < 0.05, **P < 0.01, ***P < 0.001, ****P < 0.0001

**FIGURE 3:**
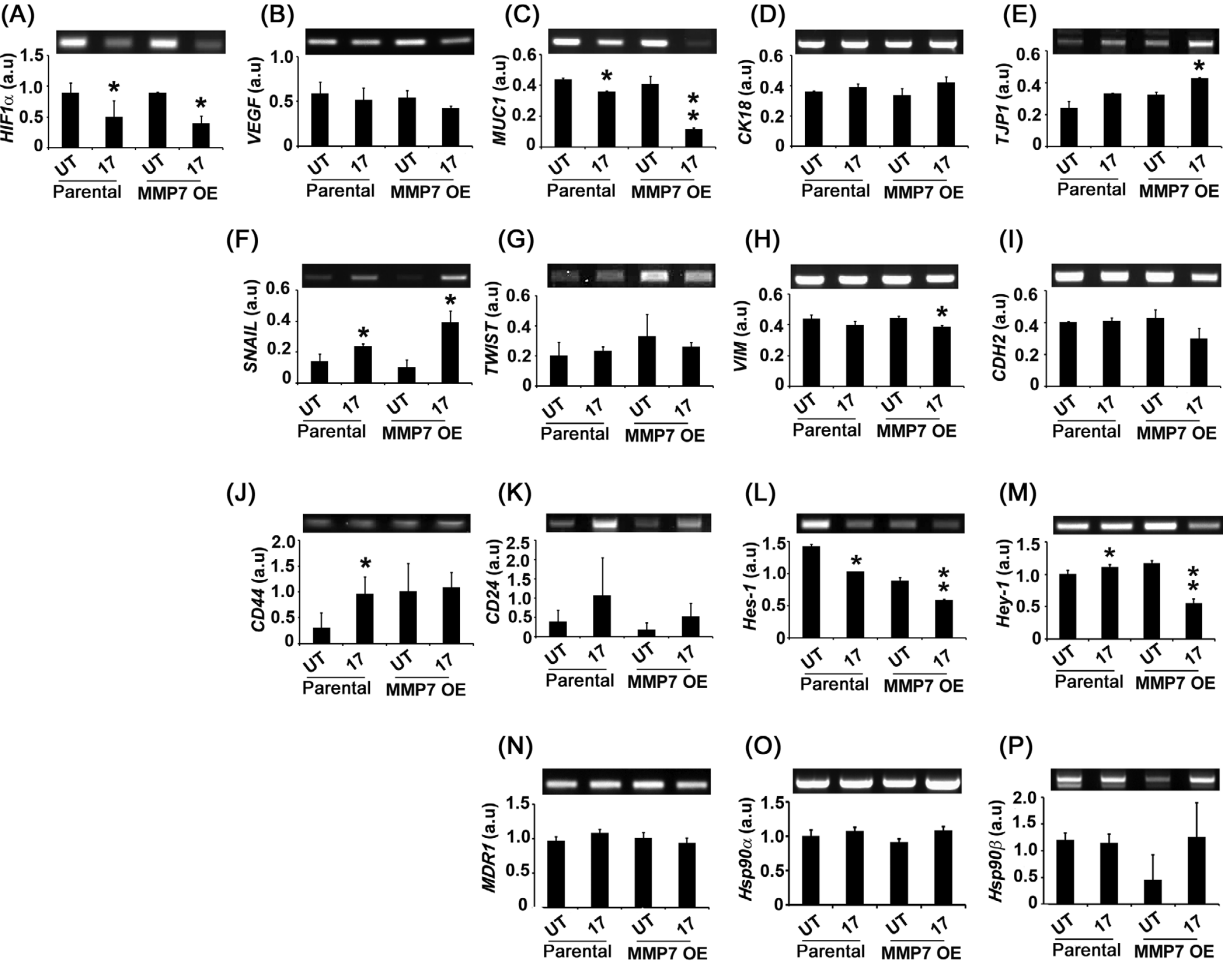
MMP7 overexpression does not correlate with acquired stemness or epithelial to mesenchymal transition (EMT). A and B, RT‐PCR analysis of hypoxia response factors, *HIF1‐α* and *VEGF* expressions. Note that 17AAG treatment decreasing the expression levels of *HIF1‐α*, but not *VEGF*. C‐E, RT‐PCR analysis of epithelial markers, *MUC1*, *CK18*, and *TJP1* expressions. Note *MUC1* expression is being decreased by 17AAG treatment in MMP7 OE cells. F‐I, RT PCR analysis of mesenchymal markers, *SNAIL*, *TWIST*, *VIM*, and *CDH2* expressions. Note that 17AAG treatment increased the expression of *SNAIL*. J and K, RT‐PCR analysis of stem markers *CD24* and *CD44* expressions. Note that 17AAG treatment increasing the expressions of *CD24* and *CD44*. L and M, RT‐PCR analysis of pluripotency markers *Hes‐1* and *Hey‐1* expressions. Note decreased pluripotency with 17AAG treatments. N‐P, RT‐PCR analysis of *MDR1*, *Hsp90α*, and *Hsp90β*. The significance values calculated from Student's *t*‐test and represented as, * *P* < 0.05, ** *P* < 0.01

**FIGURE 4:**
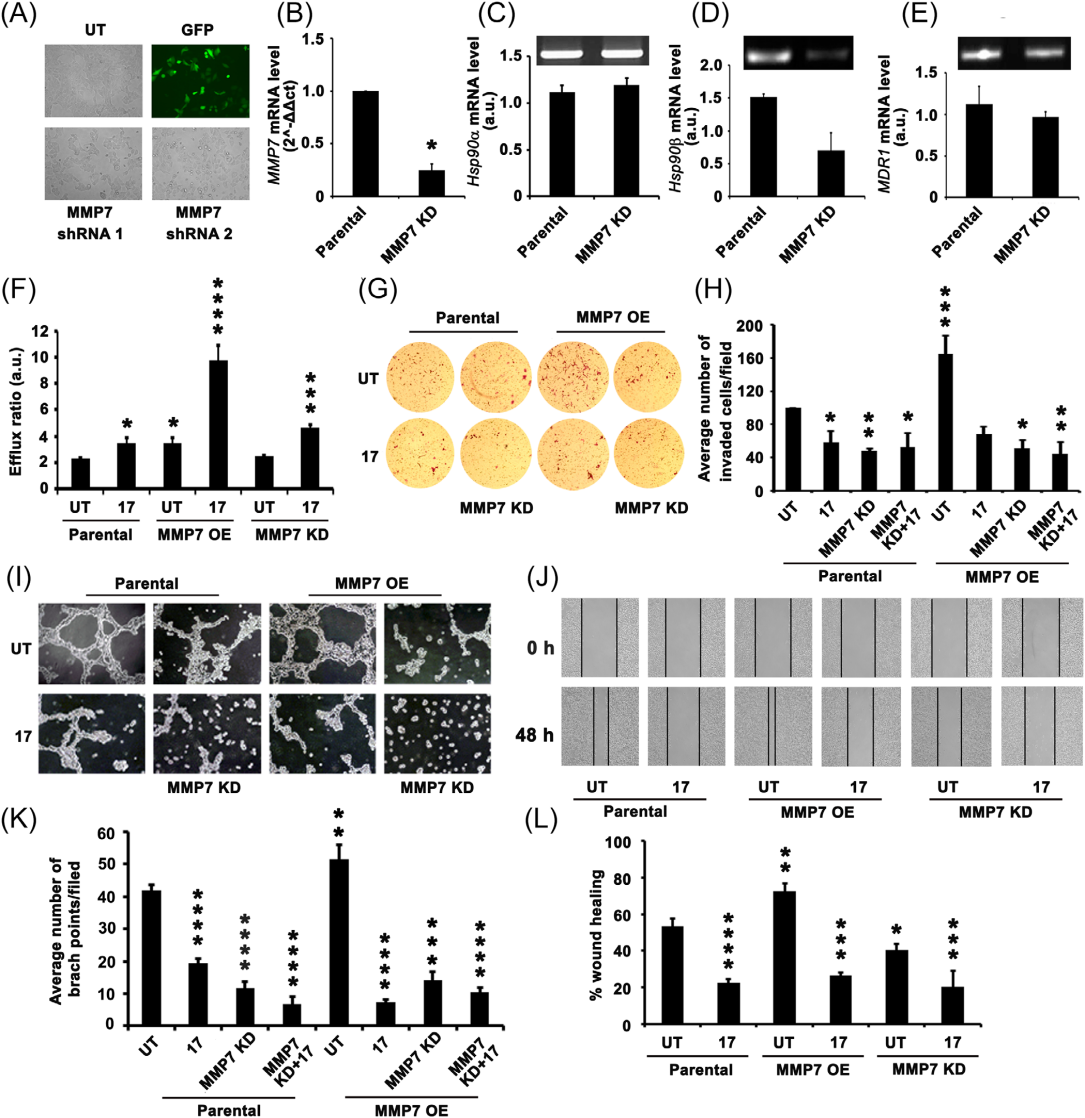
MMP7 KD decreases drug efflux activity and metastatic potential of tumor cells. A, Cell morphology after transfection with MMP7 shRNA, GFP is used as a positive control. B, Quantitative PCR analysis of MMP7 expression in Parental and MMP7 KD cells. C‐E, RT‐PCR analysis of *Hsp90α*, *Hsp90β*, and *MDR1* expressions in Parental and MMP7 KD cells, respectively. Note MMP7 KD decreasing the expression levels of *Hsp90* and *MDR1*. F, Rh123 efflux activity assay showing decreased efflux activity in MMP7 KD cells compared to MMP7 OE cells. G, Trans‐well migration assay showing decreased invasive ability of MMP7 KD cells and is comparable with Parental cells which is furthered in response to 17AAG treatment. H, A statistical representation of G. I, Angiogenesis or tube formation assay of cells grown on 3D matrix. Note that MMP7 KD decreasing the number of branch points. K, A statistical representation of I. J, The *in vitro* scratch wound heal assay. Note that MMP7 KD decreasing the wound healing property of cells compared to Parental cells which is furthered in response to 17AAG treatment. L, A statistical representation of J. The values (mean ± SD) represented in figures, B, F, H, K, and L are average values obtained from three independent experiments. The significance values calculated from one‐way ANOVA and represented as, *P < 0.05, **P < 0.01, ***P < 0.001, ****P < 0.0001

**FIGURE 5:**
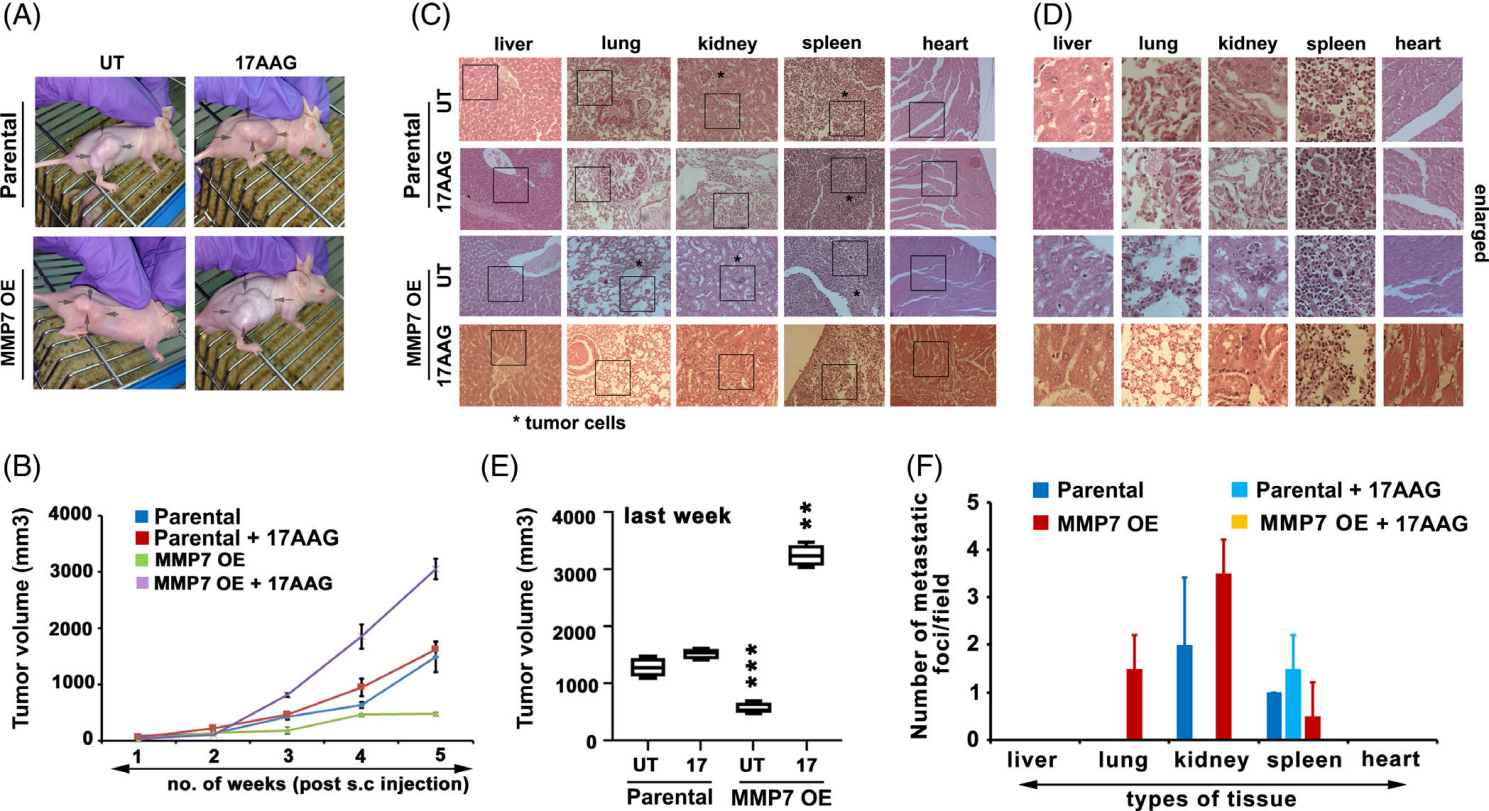
MMP7 OE cells show new homing properties, which is inhibited by 17AAG treatment. A, The mice xenografts of Parental and MMP7 OE cells. The nude mice were subcutaneously (s.c.) injected with Parental and 17AAG treated tumor cells and monitored for solid tumor growth. Note increased solid tumor growth in MMP7 OE cells upon 17AAG treatment. B & E, A statistical representation of s.c. tumor volume (mm^3^) over a period. Note that 17AAG increasing the solid tumor growth at the site of injection. The values (mean ± SD) represented are from three independent experiments and every time a minimum of six animals were used. C, Hematoxylin and Eosin (H & E) stained histological sections of various organs. The metastasized tumor cells are shown with * mark. The boxes indicate areas used for enlarged representation in D. The organs from s.c. injected xenograft bearing animals were collected and subjected to microtome sectioning and H & E staining and analyzed under the microscope (10× magnification, enlarged images of C). F, A statistical representation of the average number of metastatic foci from five different fields, C. The significance values calculated from one‐way ANOVA and represented as, *P < 0.05, **P < 0.01, ***P < 0.001

## 3 RESULTS

### 3.1 The drug‐resistant cells display induced MMP expression and heat shock response upon Hsp90 inhibition

The MMPs play a central role in tumor evolution, primarily through tumor metastasis.^29^ Further, MMP7 is considered for the prognostic assessment as well as for the therapeutic outcome.^30^ Therefore, to understand whether acquired drug‐resistance correlates with MMPs expression, we have examined the expression levels of a spectrum of major MMPs in the drug‐resistant cells (data not shown). We observed a significant increase in MMP7 expression in response to 17AAG treatment both in the Parental as well as in drug‐resistant (KBvb) phenotypes (Figure 1A). However, we could not see any alterations in cellular MMP7 protein levels suggesting that activated protease might have secreted out to serve its extracellular functions (Figures 1B and S3). In agreement with our earlier findings, we observed enhanced drug efflux activity in response to 17AAG treatment (Figure 1C). Further, we also demonstrated that P‐glycoprotein (P‐gp) majorly contributes to the acquired drug resistance, therefore, in the present study, the drug efflux activity that we mentioned is pertinent to P‐gp activity.^23^

Since induced stress response is grossly implicated in cellular adaptations that make tumor cells to evolve,^31^ we have examined the heat shock response or stress response through induced Hsp synthesis in presence and absence of quercetin, a drug that interferes with the intracellular stress response.^26^ In agreement with previous reports that Hsp90 inhibitors can induce stress response *via* the feedback loop mechanism by inducing the transcription of heat shock genes,^23^ we have observed induced expressions of *Hsp70*, *Hsp90α*, and *Hsp90β* in both Parental and KBvb cells in response to 17AAG treatment. However, quercetin could decrease the basal Hsp‐gene transcription; however, it did not interfere with Hsp‐gene transcription triggered by 17AAG treatment (Figure 1D‐F).^23^ Interestingly, in agreement with the previous study, quercetin treatment itself triggered the drug efflux activity (Figure 1G)^23^ and did not interfere with MMP7 expression (Figure 1H). These studies have confirmed that MMP7 expression is HSF1 independent (Figure 1I). Since we speculated that extracellular Hsp90 regulates the functions of secreted MMP7, we examined the secretion of Hsp90. Since we wanted to correlate enhanced drug resistance to MMP7, we have used double resistant cells, KBvbcp. However, we used KBvb cells for MMP7 overexpression in our subsequent studies. We observed increased secretion of Hsp90α in drug‐resistant cells, but not in Parental cells (Figure 1J). Subsequently, we correlated expression studies with MMP7 activity, where phorbol myristate acetate (PMA) was used to stimulate MMP7 activity. We observed constitutively enhanced MMP7 activity in all the treatments. MMP7 is upstream of MMP9 and MMP2 that are extensively implicated in tumor metastasis. Hence, we examined for the in‐gel activities of MMP7 in comparison with MMP9 and MMP2 and found that only upon PMA stimulation, the MMP7 activity correlated with MMP9 activity (Figure 1K).

### 3.2 The MMP7 OE potentiates the drug efflux activity and metastatic potential of tumor cells, however, is more sensitive to Hsp90 inhibition

MMPs implicated in tumor metastasis; thus are also considered as cancer biomarkers.^4^, ^32^ Since we observed induced drug efflux activity correlating with increased MMP7 expression, we asked whether enforced MMP7 expression alone can promote both drug efflux activity and tumor progression. Towards this, MMP7 full‐length cDNA was cloned in an expression system, transfected into KBvb cells, and stable KBvb‐MMP7 OE cells were prepared and used for drug efflux, cell cycle progression, and metastasis analyses. The MMP7 OE cells showed enhanced proliferative potential and decreased doubling time (Figure 2A). Compared to Parental cells, MMP7 OE cells showed a marginal increase in drug efflux activity, which significantly increased in response to 17AAG treatment (Figure 2B). The enhanced proliferative potential, however, did not correlate with increased cyclin D1 (*CCND1*) expression, but decreased in response to 17AAG (Figure 2C). The drug response mechanisms of cells grown under 2D culture conditions may vary on 3D culture conditions. The KB cells exhibited tube formation on 3D culture conditions, whereas MMP7 OE has increased the number of branch points indicative of the enhanced angiogenic potential of tumor cells. Interestingly, both Parental and MMP7 OE cells inhibited metastasis in response to 17AAG treatment (Figure 2D,E). Since the scratch wound heal assay not only indicate the migratory potential of cells in culture but also provide information on their proliferative ability, we performed scratch wound heal assay. While MMP7 OE cells showed enhanced migratory potential, it was inhibited by 17AAG treatment (Figure 2F,G). Subsequently, we also have examined the invasive potential of MMP7 OE cells in comparison with the Parental cells and found that MMP7 OE cells exhibited enhanced invasive potential; however, similar to Parental cells, they also exhibited decreased invasive potential in response to 17AAG treatment (Figure 2H,I). Subjecting Parental and MMP7 OE cells, both untreated and 17AAG treated to cell cycle analysis, have indicated that MMP7 OE cells are more sensitive to 17AAG treatment as observed by increased subG1 and G2/M accumulation of cells (Figures 2J and S4).

### 3.3 MMP7 overexpression do not correlate with the acquired stemness or epithelial to mesenchymal transition (EMT) of drug‐resistant cells

The acquired stem‐like features promote drug resistance and metastatic potential of tumor cells.^33^ Hence, cells grown on 3D cultures were used for the examination of key stem cell and pluripotency marker gene expressions. Since MMP induced cell proliferation is linked to their ability to respond through hypoxia,^34^ hence we examined whether MMP7 OE influences hypoxia response. While MMP7 OE is not affecting *HIF‐1α* expression, 17AAG treatment has decreased its expression in both the phenotypes (Figure 3A). Despite decreased *HIF‐1α* expression with 17AAG, the *VEGF* expression was unaffected (Figure 3B). Considering that altered MMP expressions may influence cell fate, we have examined for the epithelial (*MUC1*, *CK18*, and *TJP1*) and mesenchymal (*SNAIL*, *TWIST*, *VIM*, and *CDH2*) gene expressions. Only *MUC1* expression was decreased by 17AAG in the overexpression background (Figure 3C). The *CK18* and *TJP1* expressions showed a marginal increase in response to 17AAG treatment (Figure 3D,E). The expressions of *VIM* and *CDH2* were unaltered either by MMP7 OE or by 17AAG treatment (Figure 3H,I). Whereas *SNAIL* expression was increased by 17AAG and *TWIST* expression was increased by MMP7 overexpression (Figure 3F,G).

Since there are no alterations in either epithelial or mesenchymal gene expressions, we want to examine their stem and pluripotent status. MMPs are implicated in cell fate decisions,^35^ hence we examined for the expressions of *CD24* vs *CD44* (for stemness) and *Hes‐1* vs *Hey‐1* (for pluripotent state) expressions. Both MMP7 OE and 17AAG treatment‐increased *CD44* expression, whereas only 17AAG treatment‐induced *CD24* expression (Figure 3J,K). In contrast, the pluripotent state of cells (*Hes‐1* and *Hey‐1*)was decreased by both MMP7 OE and 17AAG treatment (Figure 3L,M). The MMP7 overexpressing cells responded to 17AAG and showed a marginal decrease in MDR1. However, the expressions of Hsp90α and Hsp90β expressions are not affected (Figure 3N‐P).

### 3.4 MMP7 KD decreases the drug efflux activity of tumor cells

The heat shock protein expression is correlated with both the aggressiveness of the tumor, which can be mediated by MMPs.^36^, ^37^ To examine the effect of MMP7 knockdown (hereafter called MMP7 KD), KBvb cells were transfected with *shRNA MMP7* and used for further studies (Figure 4A,B). We have used scrambled *shRNA* for initial evaluation and observed no difference between Parental and scrambled shRNA, hence represented the data pertinent to Parental vs *MMP7 shRNA*. First, we examined the effect of MMP7 KD on *Hsp90* isoforms and *MDR1* expressions and found that MMP7 KD does not affect *Hsp90α* (Figure 4C) and *MDR1* (Figure 4E) expressions, but interferes with *Hsp90β* expression (Figure 4D). Next, we examined whether MMP7 KD affects drug efflux activity. The MMP7 KD cells, though, exhibited a similar drug efflux activity like Parental cells, a significant increase in response to 17AAG treatment was observed. Interestingly, we observed an almost 2‐folds increase in efflux activity in the MMP7 OE background in response to 17AAG treatment (Figure 4F). Subsequently, we examined the metastatic potential of MMP7 KD cells. Earlier, we observed enhanced drug efflux activity and cell invasion in response to MMP7 OE (Figure 2I), while MMP7 KD decreasing the invasive potential of cells either alone or in response to 17AAG treatment (Figure 4G,H). Since KBvb cells exhibited tube formation on a 3D matrix (Figure 2D,E), we have compared the tube forming ability of MMP7 OE cells with MMP7 KD cells. We observed that MMP7 KD and its combination with 17AAG have significantly decreased the tube forming ability of cells, as shown by the decreased number of branch points (Figure 4I,K). Finally, we also examined the wound closure ability of cells *in vitro*. The wound closure ability of cells indicates both proliferative as well as the migratory potential. Cells exhibited high proliferative potential and wound closure upon MMP7 OE. However, both MMP7 KD and 17AAG treatments have significantly decreased wound closure ability (Figure 4J,L).

### 3.5 The MMP7 OE cells exhibit altered homing properties, which is inhibited by 17AAG treatment

Cancer treatment is challenging due to altered homing properties of cells *in vivo* in response to micro‐environmental ques. While the increased stem cells are attributed in facilitating altered homing properties, understanding tumor homing properties may help in developing targeted cancer treatments.^38^, ^39^ Towards understanding whether MMP7 OE cells acquire new homing properties, the nude mice xenograft experiments were performed. The Parental and MMP7 OE cells were injected subcutaneously into the pelvic region of male nude mice were monitored for 6 weeks for the localized subcutaneous (s.c.) tumor growth. Cells pretreated with 17AAG were used for s.c. injections to understand the influence of 17AAG on tumor growth. We observed an increase in s.c. tumor growth in Parental cells in response to 17AAG treatment. However, MMP7 OE cells showed a decrease in s.c. tumor growth, which was increased in response to 17AAG treatment. (Figure 5A, B, and E). Despite cells exhibiting increase invasion and migration (Figure 4), we observed decreased s.c. tumor volume in response to MMP7 OE, suggesting the possibility of tumor invasion. Hence, we have examined the tumor metastasis in different potential organs. The KBvb cells showed tumor metastasis in the kidney and spleen. However, MMP7 OE cells showed tumor metastasis in the lung, which is in addition to the kidney and spleen. While 17AAG treatment of Parental cells restricted tumor metastasis to spleen, 17AAG treatment of MMP7 OE cells completely inhibited the tumor metastasis. Our results demonstrate that MMP7 can influence the homing properties of tumor cells, and 17AAG treatment inhibits (Figure 5C,D). The tumor volume comparison between different s.c. injected animals before sacrificing indicates that 17AAG treatment significantly increases the s.c. tumor volume in MMP7 OE cells (Figure 5E). The number of metastatic foci were found to be maximum in lung, kidney, and spleen of mice injected with MMP7 OE cells, however, was inhibited by 17AAG treatment (Figure 5F).

## 4 DISCUSSION

The tumor spread is inhibited if tumor cells fail to reach new locations through their metastatic potential.^40^ MMPs facilitate angiogenesis and tumor metastasis by digesting the extracellular matrix (ECM), enabling tumor cells either to form new blood vessels or to invade through the existing blood vessels to reach new locations.^22^ The present study designed to address the long‐standing debate whether the acquired drug resistance and the metastatic potential of tumor cells are linked or work independently. We chose MMP7 among several MMPs examined in our laboratory since upregulated MMP7 expression correlated with enhanced drug efflux activity. We demonstrated that MMP7 facilitates both drug efflux activity and metastasis of tumor cells, however, in coordination with the cancer chaperone Hsp90. By translating our *in vitro* findings *in vivo*, we display that Hsp90 links MMP7 with the drug efflux activity, thus adds to the list of molecules to be considered for treating the drug adapted metastatic tumor cells.

The MMPs, in addition to digesting the ECM was also reported to have additional cellular functions; thus, they appear to be critical regulators of specific cell signal pathways.^29^, ^41^, ^42^ MMPs majorly implicated in tissue remodeling,^1^ but in the recent, interest on these proteinases have increased due to their involvement in disease progression, especially cancer. The current chemotherapeutic interventions suffer from limitations imposed by the acquired multidrug resistance of cancer cells. Although there was an un‐debated understanding that drug‐resistant cells are highly metastatic, yet no conclusive mechanisms are established. MMP7 is implicated in the aggression of cancer cells,^41^ thus is also presumed to play a vital role in the drug resistance of cancer cells. Strengthening this, we have observed induced expression of MMP7 in response to therapeutic challenge mediated by 17AAG treatment. Since major MMPs, MMP2 and MMP9 functions in Hsp90‐dependent manner, we used Hsp90 inhibitor, 17AAG, to understand the cross‐talk if any between MMP7 and Hsp90. We reported earlier that cancer cells that exhibit resistance to Hsp90 inhibition might become aggressive when challenged with Hsp90 inhibitors.^23^, ^43^ Although a direct link between MMP7 and Hsp90 could not be established from this study, we were able to demonstrate coordinated functions of Hsp90 and MMP7 in regulating the drug efflux activity. However, the increased MMP7 expression in response to 17AAG is demonstrated to be independent of the conventional feedback loop mechanism.

The induced metastatic potential of cells is now linked to MMP7 expression since MMP7 KD cells showed decreased metastatic potential. However, the induced drug efflux activity observed only in response to 17AAG treatment, despite MMP7 OE. Some studies indicate that therapeutic stimulation of metastatic/drug‐resistant cells stimulates the endogenous population of cancer stem cells or stimulates cancer cells to behave like cancer stem‐like cells. Earlier, we showed that metastatic breast cancer cells in response to 17AAG treatment exhibit incomplete epithelial to mesenchymal transition, thus exhibit inappropriate cell phenotypes, leading to altered homing properties.^44^ Therefore, we hypothesize that the inability of cancer cells to exhibit stem‐like characteristics, may sensitize them to 17AAG treatment and metastasis inhibition. In the present, strengthening our hypothesis, we observed indistinct ratios of CD44 and CD24, being unable to drive cellular differentiation (incomplete EMT). However, the increased proliferative ability with MMP7 OE thus suggests that MMP7 facilitates tumor spread but no self‐renewal.

Our earlier *in vitro* findings with drug‐resistant cells have indicated that inhibiting metastasis will not interfere with the drug efflux activity.^23^ To further establish, we have examined the *in vivo* behavior of tumor cells using the xenografts. We observed that 17AAG treated cells restrained from metastasis and show increased but localized s.c. tumor growth. Subsequent histology studies have indicated that MMP7 OE cells acquire new homing properties. However, they respond to 17AAG treatment and show metastasis inhibition. MMPs have first gained attention due to their correlation with unfavorable conditions during the cancer progression, and thereon targeting MMPs to interfere with tumor metastasis has emerged. However, the current MMP inhibitors aim the conserved catalytic site, thus lacks the individual MMP specificity and selectivity. It may be the reason MMP targeting could not reach fruition for treating tumors.^45^ The success stories have emerged after developing molecular tools for specific MMP inhibition; thus MMP inhibitors could enter the phase III clinical trials.^45^, ^46^, ^47^ Further, understanding the structural organization of MMPs may help in identifying regions that are not conserved but required for the proteinase activity. Towards this, the allosteric and exosite inhibitors of MMPs are recommened.^47^ Since MMP inhibitors are in the clinic and Hsp90 inhibitors are awaiting for the clinical appraisal, our study provides novel insights for drug‐resistant metastatic cancer treatment using Hsp90 inhibitors.

## CONFLICT OF INTEREST

The authors declare the conflict of interest as none.

## AUTHORS CONTRIBUTIONS

All authors had full access to the data in the study and take responsibility for the integrity of the data and the accuracy of the data analysis. *Conceptualization*, A.S.S.; *Methodology*, P.K., S.S., A.S.S.; *Investigation*, P.K., S.S.; *Formal analysis*, P.K., S.S., A.S.S.; *Writing—original draft*, P.K., A.S.S.; *Writing—review and editing*, P.K., A.S.S.; *Supervision*, *Project Administration and Funding Acquisition*, A.S.S.

## ETHICS STATEMENT

This study carried out with the guidelines and recommendations of care and use of Laboratory Animals of the National Institute of Health (NIH, Bethesda). The animal handling procedures were approved by the Institutional Animal Ethics Committee (IAEC) at CSIR for Cellular and Molecular Biology (Project Reference NO. 1/2018). Efforts made to minimize animal suffering in the entire procedure.

## Supporting information


**Data S1** Supporting Information

**Figure S1** The DNA sequence analysis of MMP7 OE recombinant plasmid. Both the subject (NM_002423.5) and query sequences are subjected to BLAST analysis and represented.

**Figure S2** The sequence analysis of shRNA recombinant plasmid. Both subject (in‐house designed) and query sequences are represented. The red color indicates shRNA.

**Figure S3** Immunoblot analysis of total cell lysate and the condition medium collected from parental, MMP7 OE and MMP7 KD cells. Note increased secretion of Hsp90 correlating with increased expression of MMP7 in the condition medium. Note that equal amounts of TCL and CM are loaded for comparison. TCL: total cell lysate; CM: condition medium.

**Figure S4** Graphical representation of DNA content analysis obtained from FACS. The untreated and drug treated cells were subjected to fluorescence activated cell sorting analysis. Each phase of cell cycle was represents as subG1, G1, S, and G2/M. Note Hsp90 inhibition showing transient cell cycle arrest, but not cytotoxicity. The statistical representation of DNA content analysis can be found in Figure 2.

## Data Availability

The data will be made available on request
