# Supplementary material for: The matrix metalloproteinase 7 (MMP7) links Hsp90 chaperone with acquired drug resistance and tumor metastasis
Source: Cancer Rep (Hoboken). 2020 Aug 6;5(12):e1261. doi: 10.1002/cnr2.1261 (PMC9780424; doi:10.1002/cnr2.1261)
Supplement: Supplementary file 1 — Data S1 Supporting Information [file CNR2-5-e1261-s002.docx]

**Supplementary data**

**Collection of conditioned medium**

The cells (4x10^5^)were grown in the complete medium in a 6-well culture plate, after 6 h, 5 mL of fresh incomplete medium was added and incubated for 24 h at standard culture conditions. The medium was stored at -80°C for overnight, followed by the tube containing media was dipped into liquid nitrogen before freeze-drying. The freeze dried medium was removed and re-suspended in 100 μL of 1X SDS Laemmli buffer. Then 20 μL of each sample was electrophoresed on 12% SDS gel and transferred onto the nitrocellulose membrane. The membrane was blocked with 5% BSA in TBST and incubated with primary antibodies to MMP7 (1:2000; ab176325, Abcam) and Hsp90α/β (1:1000; SC-7947, Santa Cruz Biotechnologies) followed by incubation with HRP-conjugated secondary antibody (1:10000; 11500694001, Sigma-Aldrich), each 1 h at room temperature. The luminescence was detected by the BM chemiluminescence detection kit (Roche, #11520709001).

**Figure S1:** The DNA sequence analysis of MMP7 OE recombinant plasmid. Both the subject (NM_002423.5) and query sequences are subjected to BLAST analysis and represented.

**Figure S2:** The sequence analysis of shRNA recombinant plasmid. Both subject (in-house designed) and query sequences are represented. The red color indicates shRNA.

**Figure S3:** Immunoblot analysis of total cell lysate and the condition medium collected from parental, MMP7 OE and MMP7 KD cells. Note increased secretion of Hsp90 correlating with increased expression of MMP7 in the condition medium. Note that equal amounts of TCL and CM are loaded for comparison. TCL: total cell lysate; CM: condition medium.

**Figure S4:** Graphical representation of DNA content analysis obtained from FACS. The untreated and drug treated cells were subjected to fluorescence activated cell sorting analysis. Each phase of cell cycle was represents as subG1, G1, S, and G2/M. Note Hsp90 inhibition showing transient cell cycle arrest, but not cytotoxicity. The statistical representation of DNA content analysis can be found in the Figure 1 of the manuscript.
