# Supplementary figures and images for: The matrix metalloproteinase 7 (MMP7) links Hsp90 chaperone with acquired drug resistance and tumor metastasis
Source: Cancer Rep (Hoboken). 2020 Aug 6;5(12):e1261. doi: 10.1002/cnr2.1261 (PMC9780424; doi:10.1002/cnr2.1261)

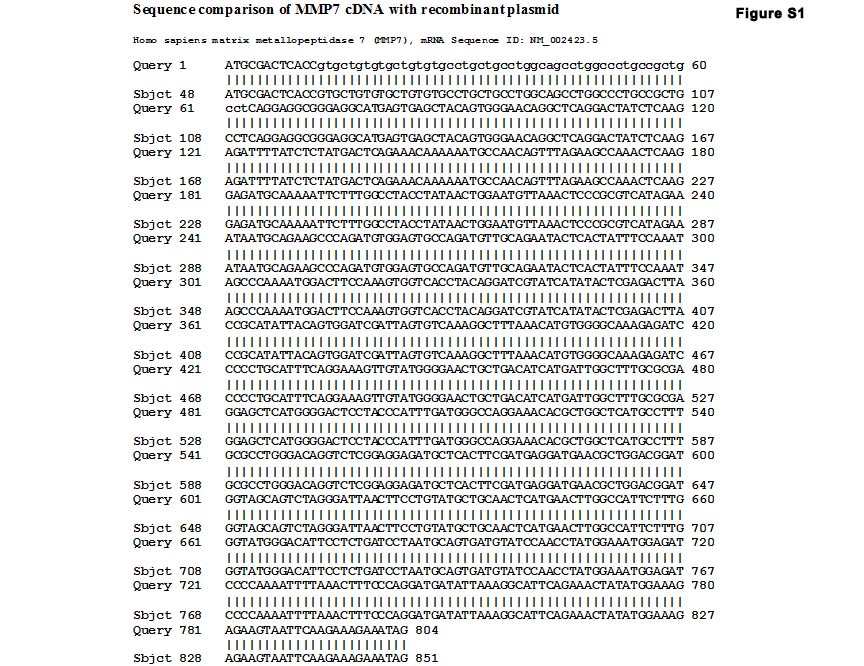

Supplement: Supplementary file 2 — Figure S1 The DNA sequence analysis of MMP7 OE recombinant plasmid. Both the subject (NM_002423.5) and query sequences are subjected to BLAST analysis and represented. [file CNR2-5-e1261-s005.jpg]

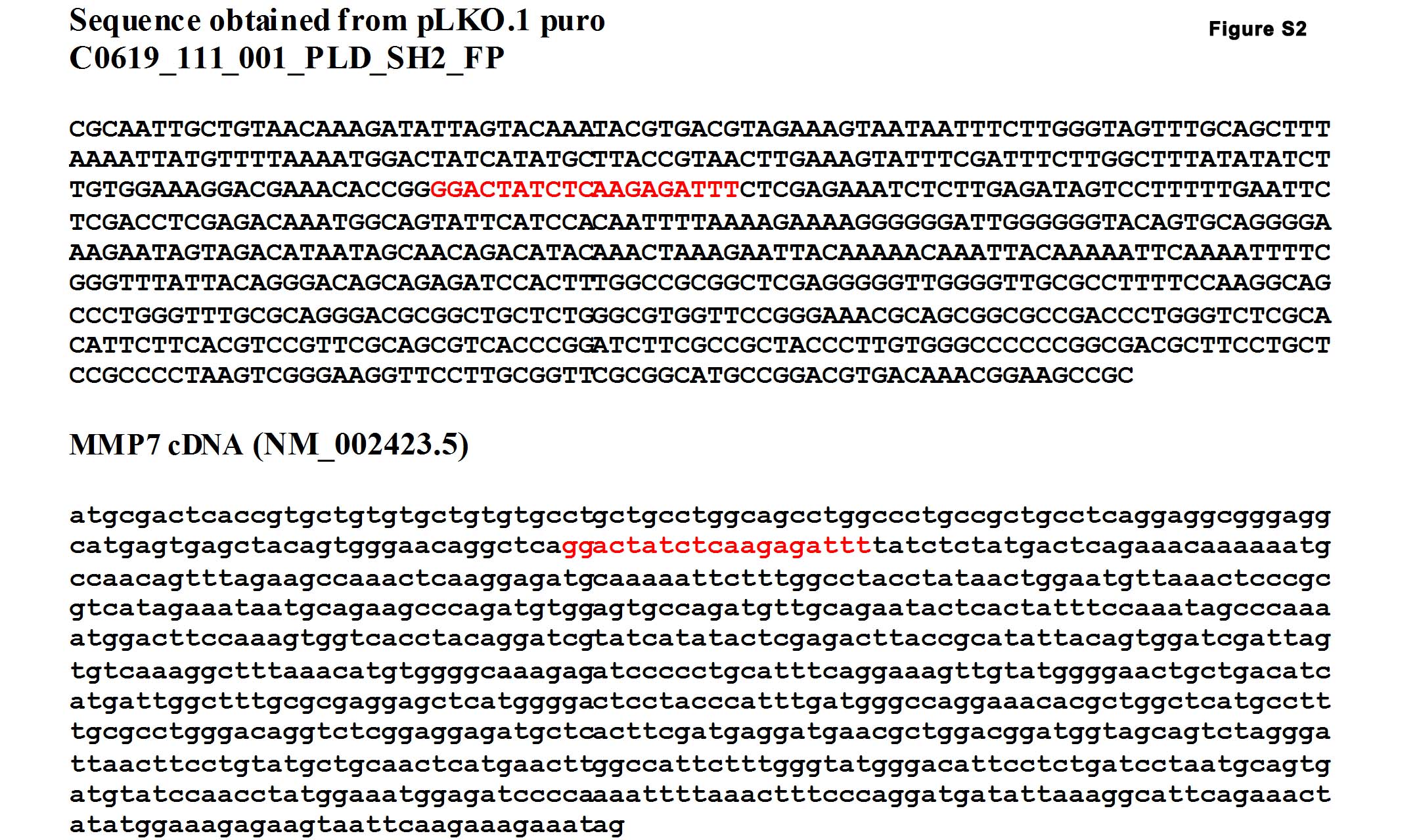

Supplement: Supplementary file 3 — Figure S2 The sequence analysis of shRNA recombinant plasmid. Both subject (in‐house designed) and query sequences are represented. The red color indicates shRNA. [file CNR2-5-e1261-s004.jpg]

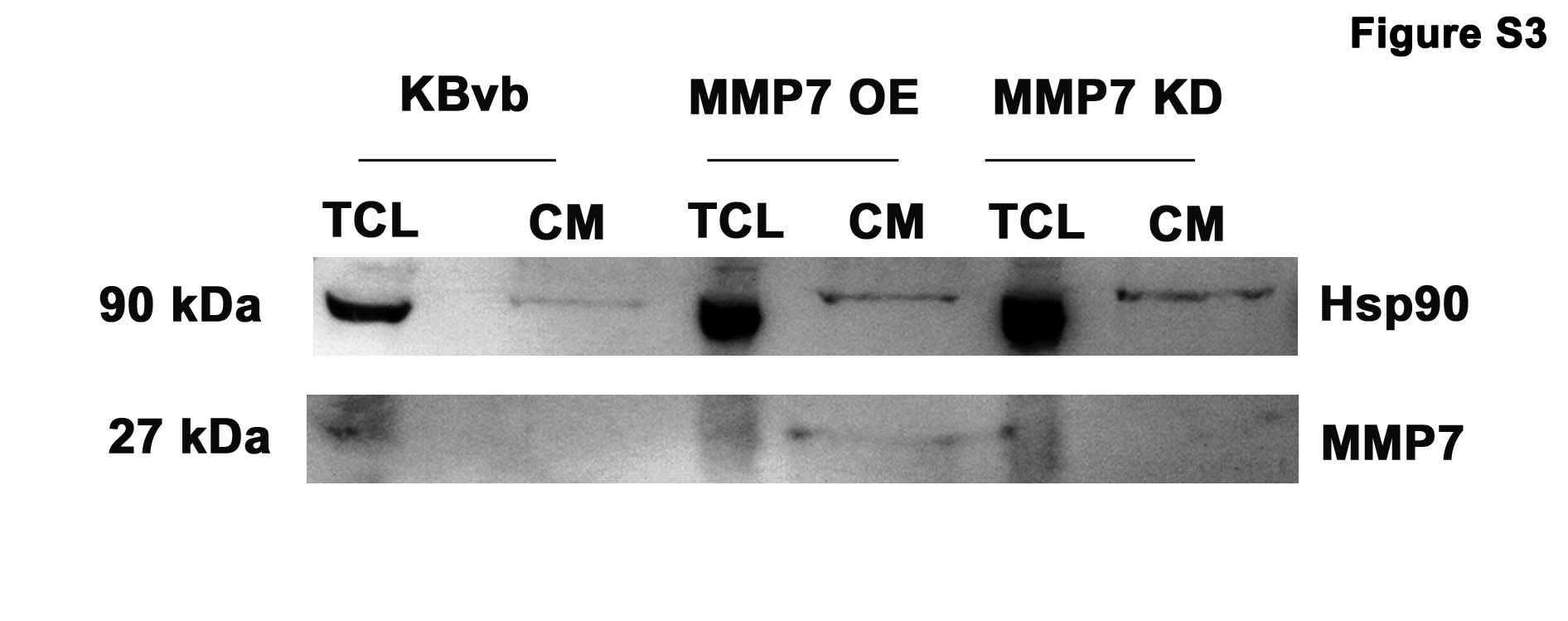

Supplement: Supplementary file 4 — Figure S3 Immunoblot analysis of total cell lysate and the condition medium collected from parental, MMP7 OE and MMP7 KD cells. Note increased secretion of Hsp90 correlating with increased expression of MMP7 in the condition medium. Note that equal amounts of TCL and CM are loaded for comparison. TCL: total cell lysate; CM: condition medium. [file CNR2-5-e1261-s003.jpg]

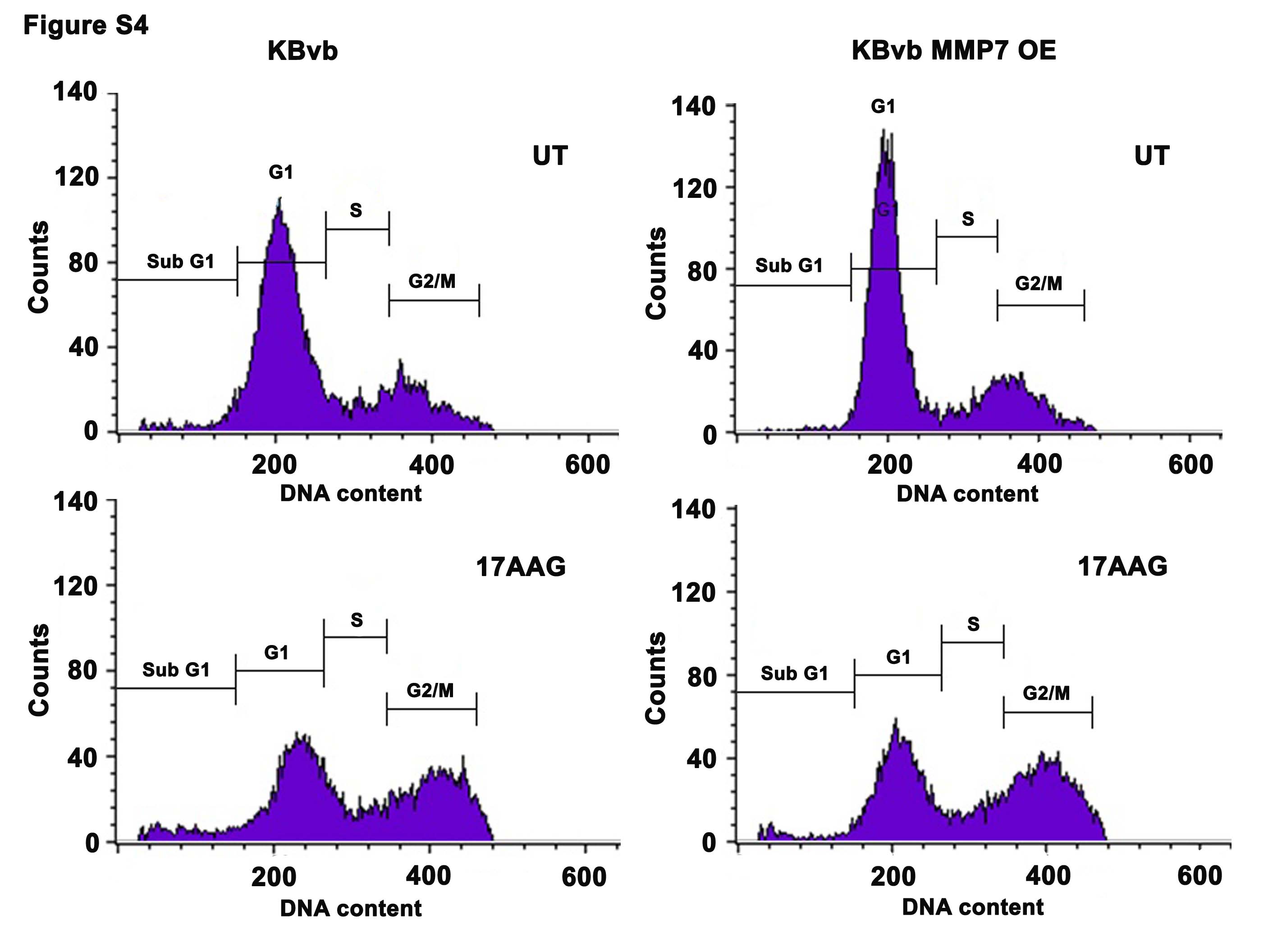

Supplement: Supplementary file 5 — Figure S4 Graphical representation of DNA content analysis obtained from FACS. The untreated and drug treated cells were subjected to fluorescence activated cell sorting analysis. Each phase of cell cycle was represents as subG1, G1, S, and G2/M. Note Hsp90 inhibition showing transient cell cycle arrest, but not cytotoxicity. The statistical representation of DNA content analysis can be found in Figure 2. [file CNR2-5-e1261-s001.jpg]
